# Personality, Cortisol, and Cognition in Non-demented Elderly Subjects: Results from a Population-Based Study

**DOI:** 10.3389/fnagi.2017.00063

**Published:** 2017-03-14

**Authors:** Sami Ouanes, Enrique Castelao, Armin von Gunten, Pedro M. Vidal, Martin Preisig, Julius Popp

**Affiliations:** ^1^Department of Psychiatry, University Hospital of LausanneLausanne, Switzerland; ^2^Department of Internal Medicine, University Hospital of LausanneLausanne, Switzerland

**Keywords:** cognition, personality, cortisol, memory, dementia, neuroticism, agreeableness, openness

## Abstract

Certain personality traits, in particular higher neuroticism, have been associated, on one hand, with elevated cortisol levels, and on the other hand, with poorer cognitive performance. At the same time, several studies highlighted the association between high cortisol and poor cognitive functioning. Here, we hypothesized that increased cortisol may be associated with poorer cognition and with certain personality traits (mainly high neuroticism), and that personality might explain the association between cortisol and cognition. A cross-sectional analysis was conducted using data from Colaus/PsyColaus, a population-based study involving residents of Lausanne, Switzerland. Salivary cortisol samples (upon waking, 30 min after waking, at 11 am and at 8 pm) along with cognitive and personality measures were obtained from 643 non-demented participants aged at least 65. Personality traits were assessed using the NEO Five-Factor Inventory (NEO-FFI). We examined the links between the cortisol Area under the Curve (AUC), the Clinical Dementia Rating Sum of Boxes (CDRSOB) and the NEO-FFI scores. No association was found between personality traits and the CDRSOB or the MMSE score, controlling for age, sex, depression, education and BMI. However, the executive functioning domain *z*-score was negatively associated with agreeableness (*p* = 0.005; slope = -0.107 [-0.181; -0.033]) and openness (*p* = 0.029; slope = -0.081 [-0.154; -0.008]) after controlling for age, sex, depression, education and BMI. The CDRSOB score was positively associated with the cortisol AUC after controlling for age, sex, BMI, education and depression, (*p* = 0.003; slope = 0.686 [0.240; 1.333]). This association remained significant after controlling for personality traits and for the interaction between personality traits and the cortisol AUC (*p* = 0.006; slope = 0.792 [0.233; 1.352]. High agreeableness and openness might be associated with poorer executive performance in later life. Increased cortisol may be associated with both specific personality traits (high extraversion, low openness) and worse cognitive performance. Increased salivary cortisol does not mediate the relationship between personality traits and cognitive impairment.

## Introduction

Certain personality traits (in particular neuroticism and to a lesser extent extraversion and openness) have been associated with Hypothalamus-Pituitary-Adrenal (HPA) axis dysregulation. Indeed, people with higher neuroticism tend to exhibit greater emotional responses to psychosocial stressors (Garcia-Banda et al., 2014). They also tend to have higher cortisol levels, likely reflecting a more pronounced stimulation of the HPA axis by psychosocial stressors (Portella et al., 2005; Yoshino et al., 2005; Gerritsen et al., 2009; Nater et al., 2010; Garcia-Banda et al., 2014; Miller et al., 2016).

However, other studies did not replicate this finding (Adler et al., 1997; Schommer et al., 1999; Ferguson, 2008) and some even reported a negative association between cortisol and neuroticism (Ballenger et al., 1983; LeBlanc and Ducharme, 2005).

Extraversion has also been associated with elevated cortisol plasma levels (Miller et al., 1999; LeBlanc and Ducharme, 2005), while agreeableness has been associated with lower salivary cortisol levels (Parent-Lamarche and Marchand, 2015).

Elevated cortisol levels have been associated with poorer cognitive performance in non-demented subjects (Lupien et al., 1994; Lee et al., 2008; Popp et al., 2009; Geerlings et al., 2015) as well as with more rapid disease progression in patients with Mild Cognitive Impairment (MCI) linked to Alzheimer’s Disease (AD) (Csernansky et al., 2006; Popp et al., 2015).

Furthermore, some personality traits have been associated with poorer cognitive performance and even with an increased risk of dementia (von Gunten et al., 2009). Indeed, in older individuals, higher neuroticism has been associated with poorer cognitive performance independently of depression (Boyle et al., 2010), and in particular with poorer episodic memory (Wilson et al., 2004). High neuroticism and low conscientiousness were associated with greater decline in executive functions (Caselli et al., 2016). In a meta-analysis examining the link between specific facets of personality and the risk of AD, individuals in the top quartile of neuroticism or the lowest quartile of conscientiousness had a threefold increased risk of incident AD (Terracciano et al., 2014).

Furthermore, previous studies examined the link between personality traits and either the HPA axis or the cognitive performance. As far as we know, no previous study scrutinized the inter-relationships between personality traits, the HPA axis and cognition.

In the light of these results highlighting that certain personality traits (higher neuroticism, in particular) might be associated with increased cortisol, that high cortisol might have deleterious cognitive effects, and that some personality traits might be associated with poorer cognition and increased risk for dementia, we hypothesized that personality traits might mediate the link between cortisol levels and cognitive impairment, in particular in episodic memory and executive functions.

## Materials and Methods

### Participants

A cross-sectional analysis was conducted using data from the first follow-up of the longitudinal population-based CoLaus/PsyCoLaus study. The methodological features of this study were already described in detail (Firmann et al., 2008; Preisig et al., 2009). CoLaus/PsyColaus included a random sample of 6734 subjects (age range: 35–75 years) selected from the residents of the city of Lausanne, Switzerland, between 2003 and 2007. All subjects were invited to participate at the first follow-up, which took place between 2009 and 2013. A total of 4004 subjects accepted these new physical and psychiatric evaluations which also included salivary cortisol measures. In addition, all subjects aged 65 or older (*n* = 1918) were invited to undergo a neuropsychological assessment. Information on demographic, medical, and treatment history as well as smoking and alcohol consumption was gathered using semi-structured interviews conducted by trained interviewers or self-rating questionnaires. Psychiatric/behavioral disorders were assessed using the Diagnostic Interview for Genetic Studies (DIGS) (Preisig et al., 1999). Cardio-metabolic disorders were assessed clinically and with the use of biochemical measures. The distribution of age groups, sex and geographic distributions in CoLaus/Psycholaus participants at baseline were similar to the source population (Firmann et al., 2008).

### Cognitive Assessment

A neuropsychological and clinical examination as well as an assessment of the participants’ daily living activities were performed by trained master-level psychologists blinded for the salivary cortisol levels.

The neuropsychological assessment included:

• The assessment of the global cognitive performance using the Mini Mental State Examination (MMSE) (Folstein et al., 1975), the most commonly used screening tool for global cognitive impairment. MMSE scores range from 0 to 30, a higher score indicating better performance.
• The assessment of *memory* using the Grober and Buschke Double Memory Test (DMT) (Buschke et al., 1997).
• The assessment of *verbal fluency* using the DO40 picture-naming test (Deloche and Hannequin, 1997), the letter (phonemic) and the category (semantic) fluency tasks.
• The assessment of *executive functions* including cognitive flexibility, selective attention, cognitive inhibition, and information processing speed using the Stroop Test (Stroop, 1935).
• The assessment of *visuo-spatial construction* using the figures from the Consortium to Establish a Registry for Alzheimer’s Disease (CERAD) neuropsychological test battery (Morris et al., 1988).

All of these tests and scales have been validated and are widely used in the field.

Overall cognitive and functional status was assessed using the Clinical Dementia Rating (CDR) scale, a widely used scale for the clinical staging of cognitive impairment. The CDR encompasses data about cognitive and functional performance in six domains: memory, orientation, judgment and problem solving, community affairs, home and hobbies, and personal care (Morris, 1993).

Participants with a global CDR > = 1 (defining dementia) were excluded from the current study.

The CDR Sum of Boxes (CDRSOB) was calculated.

### Personality Assessment

Personality traits were measured using the Revised NEO Five-Factor Inventory (NEO-FFI-R) in its French version validated in Switzerland (Aluja et al., 2005). The NEO-FFI-R is the short version of the revised NEO Personality Inventory (NEO-PI-R). The questionnaire measures the five main personality dimensions of the Five-Factor Model, i.e., neuroticism, extraversion, openness to experience, agreeableness, and conscientiousness. Participants were asked to respond to 60 items using a 5-point Likert scale ranging from 1 (strongly disagree) to 5 (strongly agree). For each of the five factors, a score is obtained by summing up the scores of the corresponding items (Costa, 1994).

### Cortisol Measures

Salivary cortisol has been established as a reliable indicator of circulating cortisol levels and HPA axis function (Gallagher et al., 2006). Participants used Salivette sampling devices (Sarstedt, Rommelsdorf, Germany) for saliva collection.

Four salivary samples were obtained from each participant: upon waking, 30 min after waking, at 11 am and at 8 pm. Subjects were instructed not to brush their teeth and to refrain from eating, drinking, smoking, and exercising 30 min prior to and during the self-administered sampling procedure (Kuehner et al., 2007). Subjects were also instructed to keep a saliva collection sheet where they would record adherence to the protocol including exact time of saliva collections. Until sampling had been completed, subjects stored the saliva samples at home in their freezers before returning them to the laboratory together with the saliva protocol, where they were stored at -20°C until biochemical analysis.

Free cortisol levels in the salivary samples were measured using a commercially available chemiluminescence assay (IBL, Hamburg, Germany). Inter- and intra-assay coefficients of variability were <9%.

In case of non-adherence to the protocol, missing values were analyzed using the expectation-maximization algorithm.

We calculated cortisol Area Under the Curve (AUC) using the trapezoid formula (Pruessner et al., 2003). The AUC is a commonly used measure to estimate total hormonal output over a period of time. Unlike other summary measures such as mean cortisol, the AUC captures not only the cortisol levels at the times of sampling but also changes over time (Pruessner et al., 2003).

We also calculated the cortisol awakening response (CAR) defined as the difference between cortisol 30 min after waking and cortisol upon waking.

### Other Variables

*Subjective cognitive decline* was assessed using the Cognitive Complaint Questionnaire (QPC) (Thomas-Antérion et al., 2003). The QPC is a French-language rater-administered instrument consisting of 10 yes/no questions assessing subjective cognitive changes over the previous 6 months. According to the scoring method proposed by the authors, subjective cognitive decline is considered present if the participant gave at least three positive answers to the 10 questions and/or a positive answer to question 5 and/or at least two positive answers to questions A,4,5,7,8.

*Depressive symptoms* were assessed at physical evaluation (approximately 1 year before the cognitive assessment) using the Center for Epidemiologic Studies Depression scale (CES-D) in its French version (Morin et al., 2011). CES-D scores range between 0 and 60 with higher scores indicating more depressive symptoms. Depressive symptoms were clinically re-assessed at the same time as the cognitive assessment.

Level of formal *education* was categorized into two groups: primary/secondary school education and higher education.

*Body Mass Index (BMI)* was also included as a covariate since cortisol levels might depend on BMI (Odeniyi et al., 2015).

### Ethics Statement

The CoLaus/PsyCoLaus study was approved by the Ethics Committee of the University of Lausanne and written informed consent was obtained from all participants.

### Statistical Analysis

Statistical analysis was performed using SPSS v23.0 (IBM Corp., Armonk, NY, USA).

#### Descriptive Statistics

Continuous variables were described as mean ± standard deviation, while categorical variables were described through absolute and relative frequencies.

The scores of each cognitive domain (memory, fluency, executive functions and visuo-spatial construction) were standardized (*z*-scores were calculated for the sum of each cognitive domain score).

#### Associations between Cognitive Performance and Cortisol

Hierarchical multiple linear regression models were constructed with the MMSE score as an outcome variable and with the cortisol AUC then the CAR (respectively) as independent variables, controlling for age, sex, BMI, education and CES-D score.

Afterward, each cognitive domain *z*-score was considered as a dependant variable with cortisol AUC then CAR (respectively) as independent variables, controlling for age, sex, BMI, education and CES-D score.

#### Associations between Cognitive Performance and Personality Traits

Hierarchical multiple logistic regression models were constructed with the MMSE score, then with the CDRSOB and each cognitive domain *z*-score respectively as dependent variables, and with the NEO-FFI-R trait scores as independent variables, controlling for age, sex, BMI, education and CES-D score.

#### Associations between Cortisol and Personality Traits

Hierarchical multiple logistic regression models were constructed with the cortisol AUC then with the CAR respectively as dependent variables, and with the NEO-FFI-R trait scores as independent variables, controlling for age, sex, BMI, education and CES-D score.

#### Associations between Cognitive Performance, Cortisol and Personality Traits

A three-block hierarchical multiple regression model was constructed with the MMSE score as a dependant variable. Block one included age, sex, BMI, education and the CES-D score. Block two included the NEO-FFI-R trait scores and the interaction between NEO-FFI-R trait scores and the cortisol AUC and block three the cortisol AUC.

For each of these regression models, the unstandardized regression coefficients (B), their 95% confidence intervals (CIs), the corresponding R squares (*R*^2^) and the *p*-values are presented.

The mediation hypothesis was assessed using Sobel’s method (Sobel, 1982).

For multiple comparisons, *p*-values were adjusted according to Holm–Bonferroni’s method.

## Results

### Characteristics of the Sample

**FIGURE 1:**
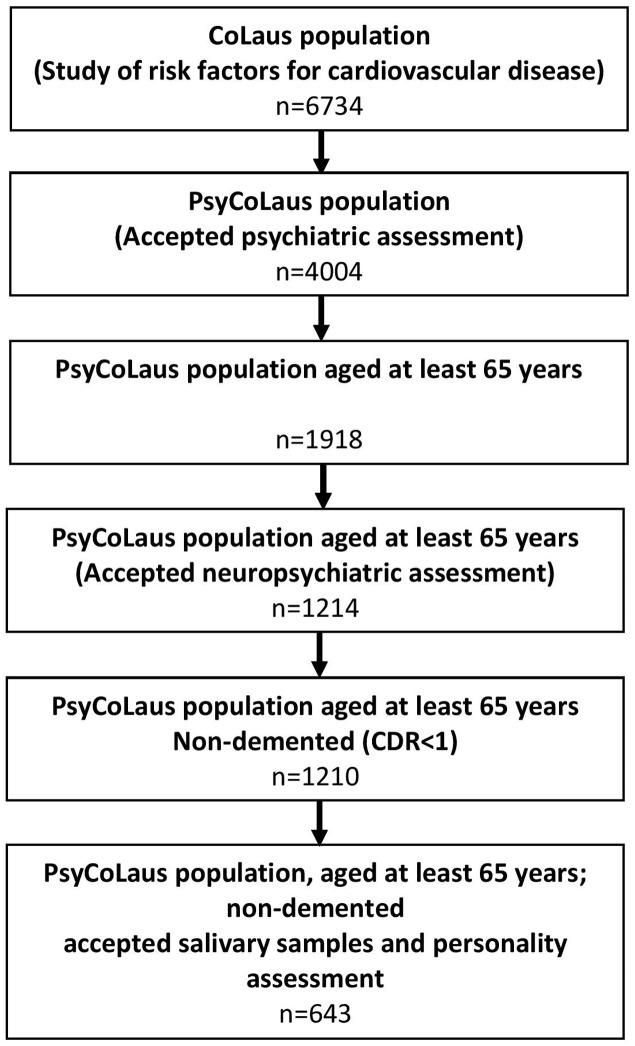
**Flow chart of the included participants**.

Of the 1918 subjects aged at least 65 at the first follow-up visit of CoLaus/PsyCoLaus, 1214 had a thorough cognitive assessment and 643 non-demented (CDR score < 1) individuals also agreed to provide salivary samples and underwent a personality assessment (33.5% of all participants in this age range) (**Figure 1**).

Compared with the whole PsyCoLaus sample aged at least 65, our sub-sample had a comparable distribution by age, gender, education level, BMI and CES-D score (**Table 1**).

**Table 1:** General characteristics of the studied samples.

	PsyCoLaus sample aged at least 65	Studied sample	*p*
*n*	1214	643	
Age, years (m ± SD)	71.6 ± 4.7	71.5 ± 4.5	0.66
Gender, % women	57.9	57.7	0.93
Education level, % higher education	40.5	38.3	0.36
BMI, Kg/m^2^ (m ± SD)	26.9 ± 4.7	26.7 ± 4.5	0.38
CES-D score (m ± SD)	10.3 ± 8.2	10.0 ± 7.9	0.45


*m, mean; SD, standard deviation; BMI, Body Mass Index; CES-D, Center for Epidemiologic Studies Depression Scale.*

Among the salivary cortisol measures, 164 (6.4% of the total number of values) were missing.

The results of the different cognitive scores, the personality assessment as well as the cortisol measures are described in **Table 2**.

**Table 2:** Cognitive, personality and cortisol profile of the studied population.

MMSE score (0–30) (m ±*SD*)	29.2 ± 1.6
CDRSOB median (P25;P75)	0.5 (1.0; 1.5)
CDR score, n(%)	
0	291 (45.3)
0.5	352 (54.7)
QPC, subjective cognitive decline present, n(%)	42 (21.0)
DMT (m ±*SD*)	
Immediate recall (0–16)	15.8 ± 1.1
Total free recall (0–48)	29.8 ± 6.9
Total cued recall (0–48)^∗^	18.0 ± 9.2
Identification (0–16)	15.9 ± 0.4
Recognition (0–48)	44.8 ± 9.4
Delayed free recall (0–16)	11.6 ± 2.6
Delayed cued recall (0–16)^∗^	4.9 ± 3.7
DO40 picture-naming test (0–40) (m ±*SD*)	39.7 ± 1.2
Semantic verbal fluency (m ±*SD*)	29.5 ± 8.1
Phonemic verbal fluency (m ±*SD*)	20.9 ± 7.8
Stroop test, correct items (m ±*SD*)	23.9 ± 0.6
Stroop dots condition (0–24)	23.9 ± 0.6
Stroop words condition (0–24)	23.9 ± 0.4
Stroop interference condition (0–24)	23.2 ± 1.9
CERAD figures (0–11) (m ±*SD*)	10.5 ± 1.0
NEO-FFI	
Neuroticism (0–48) (m ±*SD*)	17.3 ± 6.7
Extraversion (0–48) (m ±*SD*)	27.3 ± 6.0
Openness (0–48) (m ±*SD*)	29.0 ± 5.6
Agreeableness (0–48) (m ±*SD*)	33.8 ± 5.1
Consciousness (0–48) (m ±*SD*)	34.6 ± 5.4
Cortisol AUC, in μmol.h/L (m ±*SD*)	0.28 ± 0.11
CAR, in nmol/L, median (P25;P75)	6.3 (-0.5; 12.7)


*m, mean; SD, standard deviation; CDR, Clinical Dementia Rating; CDRSOB, Clinical Dementia Rating Sum Of Boxes; QPC, Cognitive Complaint Questionnaire; MMSE, Mini Mental State Examination; DMT, Grober and Buschke Double Memory Test; DO40: CERAD, Consortium to Establish a Registry for Alzheimer’s Disease; NEO Five-Factor Inventory; AUC, Area Under the Curve; CAR, Cortisol Awakening Response.*

*^∗^Cued recall scores refer to the number of additional items recalled with cues after the free recall step.*

Subjective cognitive decline was not associated with cortisol, cognition, depression or personality traits, and was not included in further statistical analysis.

**Table 3** shows the Spearman’s correlations between the Big Five traits in our population. Although the correlations were significant, they were modest, and induced no multicollinearity issues in regression models as checked by tolerance.

**Table 3:** Spearman correlations between the Big Five traits in the studied population.

		Neuroticism	Extraversion	Openness	Agreeableness	Conscientiousness
Neuroticism	ρ^∗^ *p*	-	-0.391 <0.001	-0.165 <0.001	-0.276 <0.001	-0.401 <0.001
Extraversion	ρ^∗^ *p*		–	0.271 <0.001	0.244 <0.001	0.426 <0.001
Openness	ρ^∗^ *p*			–	0.178 <0.001	0.171 <0.001
Agreeableness	ρ^∗^ *p*				–	0.320 <0.001


*^∗^ρ, Spearman’s correlation coefficient.*

### Personality Traits and Cortisol

Cortisol AUC was associated negatively with extraversion (*p* = 0.005; slope = -2.850 [-4.854; -0.846]) and positively with openness (*p* = 0.008; slope = 2.412 [0.628; 4.197]; *R*^2^ = 0.030) after controlling for age, sex, depressive symptoms and BMI.

Cortisol awakening response was negatively associated with the neuroticism score (*p* = 0.048; slope = -1.117 [-2.222; -0.011]; *R*^2^ = 0.031) after controlling for age, sex, depression, education and BMI.

### Personality Traits and Cognitive Performance

No association was found between personality traits and the CDRSOB or the MMSE score, controlling for age, sex, depression, education and BMI.

Among the cognitive domains, only the executive functioning domain *z*-score was negatively associated with agreeableness (*p* = 0.005; slope = -0.107 [-0.181; -0.033]) and openness (*p* = 0.029; slope = -0.081 [-0.154; -0.008]; *R*^2^ = 0.107) after controlling for age, sex, depression, education and BMI.

### Cognitive Performance and Cortisol

**FIGURE 2:**
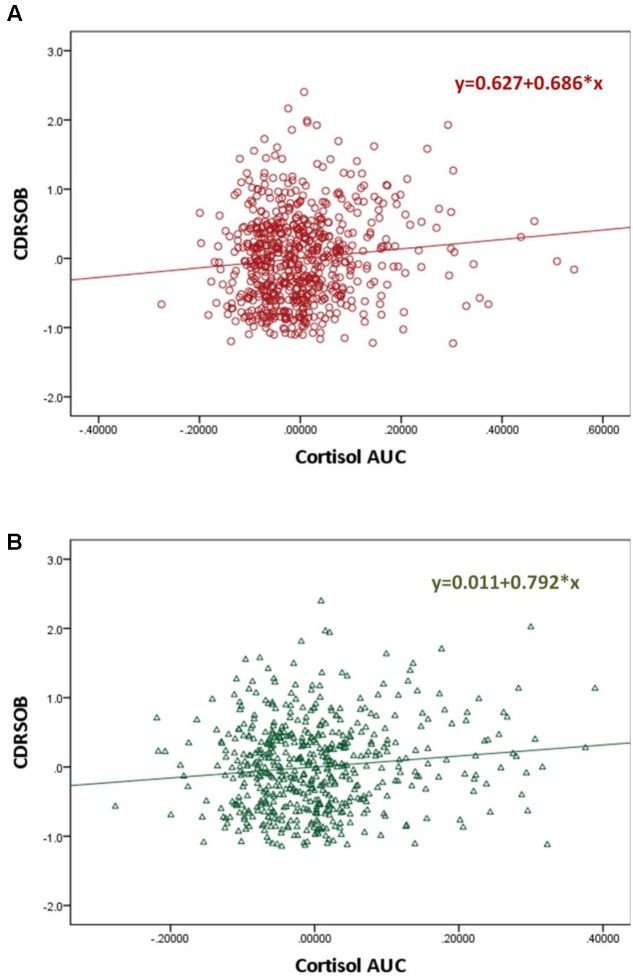
**Scatter plot graphs of partial regression analysis result for the association between the MMSE and Cortisol Area Under the Curve before** **(A)** and after **(B)** controlling for personality Big Five traits and the interaction between Big Five traits and the cortisol AUC^∗^

The CDRSOB was positively associated with the cortisol AUC after controlling for age, sex, BMI, education and depressive symptoms (*p* = 0.003; slope = 0.686 [0.240; 1.333]; *R*^2^ = 0.089) (**Figure 2**). Similarly, the MMSE score was negatively associated with the cortisol AUC, after controlling for age, sex, BMI, education and depressive symptoms (regression *p* = 0.039; slope = -1.079 [-2.106; -0.052]; *R*^2^ = -0.039) These associations became not significant, however, when the individuals with a cortisol AUC above the 95th percentile are excluded.

Among the cognitive domains, only the memory domain *z*-score was significantly associated with cortisol AUC (*p* = 0.025; slope = -0.791 [-1.485; -0.098]; *R*^2^ = 0.083). Neither the MMSE score nor any of the cognitive domain *z*-scores was associated with the CAR.

### Are Personality Traits Related to the Cortisol AUC Thereby Affecting Global Cognitive Performance?

The CDRSOB score was positively associated with the cortisol AUC after controlling for age, sex, BMI, education and depression, (*p* = 0.003; slope = 0.686 [0.240; 1.333]; *R*^2^ = 0.089). This association remained significant after controlling for personality traits and for the interaction between personality traits and the cortisol AUC (*p* = 0.006; slope = 0.792 [0.233; 1.352]; *R*^2^ = 0.107) (**Figure 2**). Sobel test for mediation was not significant.

*Post hoc* power analysis found an observed statistical power of 0.993.

## Discussion

We analyzed the relationships between personality traits, cortisol levels and cognitive performance in a large sample of non-demented subjects aged 65 years or more from the general population. While higher cortisol was associated both with some traits (low extraversion and high openness) and with poorer cognition, our results suggest that cortisol does not mediate the relationship between personality traits and cognition.

### Personality Traits and Cortisol

In our study, individuals with higher openness tended to have higher cortisol levels. This finding is in line with Bibbey et al.’s (2013) study which showed that participants who were less open had smaller cortisol reaction to stress. Yet, other studies did not find any association between openness and cortisol (LeBlanc and Ducharme, 2005; Gerritsen et al., 2009; van Santen et al., 2011). This discrepancy is likely explained by different studied populations.

Lower extraversion was associated with higher cortisol levels. Since extraversion is associated with warmth, assertiveness and positive emotions (LeBlanc and Ducharme, 2005), we might expect extravert individuals to deal better with stress and thus to have higher thresholds to activate their HPA axis. However, previous studies either showed an opposite tendency (higher extraversion associated with higher cortisol) (Miller et al., 1999; LeBlanc and Ducharme, 2005), or found no significant association (Ballenger et al., 1983; Schommer et al., 1999).

We did not find any link between neuroticism and cortisol. While this result is similar to that of some other studies (Adler et al., 1997; Schommer et al., 1999; Ferguson, 2008), neuroticism has often been associated with elevated cortisol (Bridges and Jones, 1968; van Eck et al., 1996; Miller et al., 1999; Portella et al., 2005; Yoshino et al., 2005; Gerritsen et al., 2009; Nater et al., 2010; Garcia-Banda et al., 2014; Miller et al., 2016), yet also with decreased cortisol (Ballenger et al., 1983; LeBlanc and Ducharme, 2005).

This discrepancy is probably due to different studied populations and to the effects of depression and anxiety which have been controlled for in some studies but not in others.

### Personality Traits and Cognitive Performance

We did not find any relationship between the MMSE score or the CDRSOB and personality traits. In our study, we considered all of the Big Five factors, whereas most prior studies examining the link between cognition and personality traits focused solely on neuroticism and a few additionally examined extraversion.

Higher neuroticism has been reported to be associated with lower MMSE scores in older primary care patients independently of depression (Boyle et al., 2010) as well as community-dwelling individuals aged at least 70 years independently of depression and anxiety (Jorm et al., 1993). This association might be stronger in demented subjects but seems to be also present among non-demented individuals (Jorm et al., 1993). Higher neuroticism seems to be associated with poorer episodic memory in community-dwelling individuals (Jorm et al., 1993; Meier et al., 2002; Klaming et al., 2016) as well as in patients with AD (Wilson et al., 2004). Higher neuroticism has been shown to be linked to an increased risk of incident AD dementia (Terracciano et al., 2014).

Data about the association between extraversion and cognitive measures have been scarce and discrepant. Indeed, Meier et al. found that extraversion positively correlated with episodic memory performance (Meier et al., 2002). This link was explained by more extraverted older adults being probably more stimulated and less affected by the loss of social interactions that occurs with age (Meier et al., 2002). However, others did not find any link between cognition and extraversion (Jorm et al., 1993).

A meta-analysis examining personality traits and the risk for AD showed that higher agreeableness could be a protective factor against AD (Terracciano et al., 2014).

Moreover, since agreeableness is characterized by altruism and kindheartedness, agreeable people might be more inclined to deal positively with stressors. Hence, individuals with low agreeableness are probably more prone to react negatively to stressors, in the same way as people with high neuroticism, thus explaining our finding (Parent-Lamarche and Marchand, 2015).

We found that individuals scoring higher on agreeableness or openness had poorer executive functioning. This result is in line with Ikeda et al.’s (2014) study reporting an association between higher agreeableness scores and weakened suppression of the default mode network. This network consists of the posterior cingulate cortex and precuneus, the inferior parietal cortices, and dorsal and ventral areas of the medial frontal cortex and is normally suppressed during frontal tasks (Ikeda et al., 2014).

Finally, we did not find any association between conscientiousness and cognition, while a clinical and autopsy study, found higher premorbid conscientiousness in demented patients with cerebral AD pathology compared to non-demented individuals with cerebral AD pathology (Terracciano et al., 2014).

### Are Personality Traits Related to the Cortisol AUC Thereby Affecting Global Cognitive Performance?

Specific personality traits might alter cognition and/or increase the risk for AD through increased cortisol output. Indeed, as personality traits affect the way individuals cope with stressors they might modulate the effects of the various stressors over the lifespan on the HPA axis. At the same time, elevated cortisol has commonly been associated with cognitive impairment (Lupien et al., 2007; Lee et al., 2008; Tatomir et al., 2014; Geerlings et al., 2015; Vogel et al., 2016), hippocampal atrophy (Sapolsky, 2000; Tatomir et al., 2014) and AD pathology (Green et al., 2006).

Our findings suggest that personality traits might not mediate or modify the association between cortisol and cognitive performance. Certain personality traits might lead to poorer cognitive performance, independently of cortisol, through other mechanisms including higher rates of cigarette smoking, physical inactivity, obesity and depression which in turn are known risk factors for dementia, as well as effects on inflammatory markers (particularly, IL6, CRP and leucocyte count) and neurotrophic factors. Furthermore, shared genetic liability (involving the *DYRK1A* and the APOE genes) has also been suggested as a possible mechanism for the association between specific personality traits and AD pathology (Terracciano et al., 2014).

The association we found between cortisol and cognitive performance can be explained by factors other than stress increasing cortisol output more or less depending on the personality traits. Indeed, increased cortisol is associated with the metabolic syndrome, which is tightly linked to vascular dementia and AD (Kim and Feldman, 2015). Moreover, since the hippocampus normally inhibits the HPA axis, the hippocampal atrophy associated with AD might promote the cortisol release (Geerlings et al., 2015).

### Strengths and Limitations

Major strengths of this study include its population-based design, the large sample size for this type of studies (many other studies had fewer than 100 participants) as well as the detailed neuropsychological examination. While many other studies only examined one or two personality traits, mainly neuroticism, we assessed the Big Five personality traits. A previous personality assessment allowed us to disentangle effects of pre-morbid personality traits from personality changes associated with cognitive decline.

Nevertheless, some limitations are to be acknowledged. First, the cross-sectional design does not allow us to draw any conclusions about cause-to-effect relationships. Second, only 33.5% of the participants of 65 years and older underwent both the cognitive and the personality assessments and the cortisol measures. However, the included sub-sample had a comparable distribution by age, gender, education level, BMI and CES-D score with the whole PsyCoLaus sample aged at least 65. Third, since the assessment of personality traits was cross-sectional, it does not allow us to draw any conclusions on whether the measured traits were the “stable” adult traits or the traits already modified by a potential neurodegenerative process. Indeed, personality changes seem to occur early in the course of neurocognitive disorders, at the stage of MCI (Donati et al., 2013), although these changes seem to remain moderate. Fourth, we did not collect information about perceived stress at the moment of the study. Different levels of perceived stress might have affected the cortisol levels.

## Conclusion

High agreeableness and openness might be associated with poorer executive performance. High extraversion and low openness might be associated with increased cortisol. The association between personality traits and cognitive impairment seems to be independent of increased cortisol production and its effects on cognition. Hence, specific personality traits may influence cognition through other mechanisms. According to other studies, these mechanisms may include effects on lifestyle and health related behavior including smoking, dietary habits and physical activity with consequences for cardiovascular risk and the production of cytokines and neurotrophic factors.

## Author Contributions

Conception and design of the study: SO, EC, AvG, PV, MP, and JP. Data analysis/interpretation: SO, EC, MP, and JP. Drafting the article: SO and JP. Revising the article: SO, EC, AvG, PV, MP, and JP.

## Conflict of Interest Statement

The authors declare that the research was conducted in the absence of any commercial or financial relationships that could be construed as a potential conflict of interest. The reviewer XZ and handling Editor declared their shared affiliation, and the handling Editor states that the process nevertheless met the standards of a fair and objective review.
